# Synthesis, *in vitro* antifungal evaluation and *in silico* study of 3-azolyl-4-chromanone phenylhydrazones

**DOI:** 10.1186/2008-2231-20-46

**Published:** 2012-10-04

**Authors:** Adile Ayati, Mehraban Falahati, Hamid Irannejad, Saeed Emami

**Affiliations:** 1 Department of Medicinal Chemistry and Pharmaceutical Sciences Research Center, Faculty of Pharmacy, Mazandaran University of Medical Sciences, Sari, Iran; 2 Department of Parasitology, Faculty of Medicine, Tehran University of Medical Sciences, Tehran, Iran

**Keywords:** Azole antifungals, Antifungal activity, 1 *H*-imidazole, 1,2,4-triazole, Chroman-4-one, Hydrazone

## Abstract

**Background:**

The currently available antifungal drugs suffer from toxicity, greatest potential drug interactions with other drugs, insufficient pharmacokinetics properties, and development of resistance. Thus, development of new antifungal agents with optimum pharmacokinetics and less toxicity is urgent task. In the search for new azole antifungals, we have been previously described azolylchromanone oxime ethers as rigid analogs of oxiconazole. In continuation of our work, we incorporated phenylhydrazone moiety instead of oxime ether fragment in azolylchromanone derivatives.

**Methods:**

The 3-azolyl-4-chromanone phenylhydrazones were synthesized via ring closure of 2-azolyl-2'-hydroxyacetophenones and subsequent reaction with phenylhydrazine. The biological activity of title compounds was evaluated against different pathogenic fungi including *Candida albicans*, *Saccharomyces cerevisiae*, *Aspergillus niger*, and *Microsporum gypseum*. Docking study, *in silico* toxicity risks and drug-likeness predictions were used to better define of title compounds as antifungal agents.

**Results:**

The *in vitro* antifungal activity of compounds based on MIC values revealed that all compounds showed good antifungal activity against *C. albicans*, *S. cerevisiae* and *M. gypseum* at concentrations less than 16 μg/mL. Among the test compounds, 2-methyl-3-imidazolyl derivative **3b** showed the highest values of drug-likeness and drug-score.

**Conclusion:**

The 3-azolyl-4-chromanone phenylhydrazones considered as analogs of 3-azolyl-4-chromanone oxime ethers basically designed as antifungal agents. The antifungal activity of title compounds was comparable to that of standard drug fluconazole. The drug-likeness data of synthesized compounds make them promising leads for future development of antifungal agents.

## Introduction

Recently, the incidence of life-threatening fungal diseases has dramatically increased due to the rising number of immunocompromised patients, increased use of anti-cancer drugs, and immunosuppressive therapy for organ transplantation [1]. On the other hand, the currently available antifungal drugs suffer from toxicity, greatest potential drug interactions with other drugs, insufficient pharmacokinetics properties, and development of resistance [2]. Consequently, it is urgent to develop new broad-spectrum antifungal agents with optimum pharmacokinetics and less toxicity.

Several antifungal drugs, including azoles, polyenes, echinocandins and allylamines have been developed to overcome the fungal infections [3]. However, in comparison to the large number of antibacterial drugs the number of clinically available antifungal agents is rather low. Azole antifungals such as ketoconazole, fluconazole, voriconazole, and posaconazole, which inhibit fungal cytochrome P-450 dependent 14α-demethylase, are among the most investigated compounds with respect to their clinical impact and individual mechanism of action [4]. These antifungal drugs contain imidazole or triazole heterocycles and act through a mechanism in which the heterocyclic nitrogen atom (N-3 of imidazole or N-4 of triazole) binds to the heme iron atom in cytochrome P-450 enzymes. Particularly, the triazole derivatives have a greater affinity for fungal cytochrome P-450 enzymes than for mammalian ones contributing to a favorable safety profile [5].

**Figure 1:**
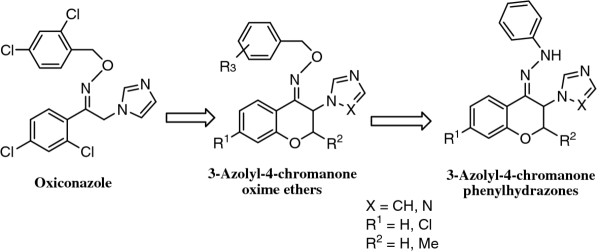
Structures of azolylchromanone oxime ethers as rigid analogs of oxiconazole and designed compounds 3-azolyl-4-chromanone phenylhydrazones.

**Figure 2:**
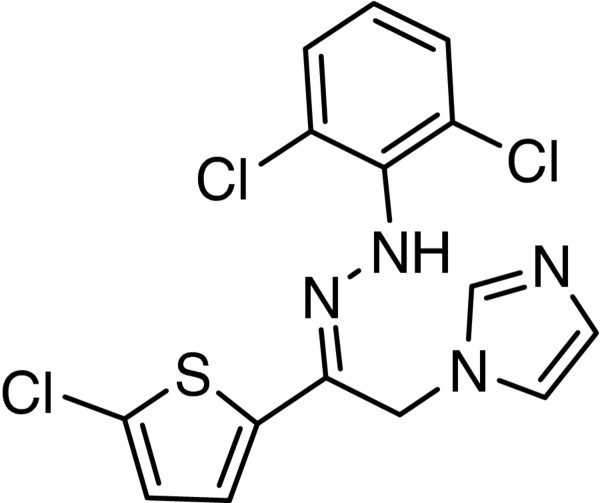
Zinoconazole, a well-known azole antifungal bearing phenylhydrazone substructure.

In the search for new azole antifungals, we have been described azolylchromanone oxime ethers (Figure 1) as rigid analogs of oxiconazole [6-8]. The chroman scaffold also found in some natural flavonoids possessing antifungal activity [9-11]. In continuation of our work, we incorporated phenylhydrazone moiety instead of oxime ether fragment in azolylchromanone derivatives (Figure 1). This modification was made on the basis of zinoconazole, a well-known azole antifungal possessing phenylhydrazone substructure (Figure 2). Thus we describe here, the synthesis and antifungal activity of 3-azolyl-4-chromanone phenylhydrazones. Furthermore, docking study and *in silico* toxicity risks and drug-likeness predictions were used to better define of title compounds as antifungal agents.

## Material and methods

### Chemistry

All chemical reagents and solvents were purchased from Merck AG Chemical (Germany) and used without further purification. The progress of reactions and the purity of compounds were checked by TLC analysis on silica gel 60 F254 plates (Merck) and visualization was done with UV light (254 nm). Melting points were determined in open glass capillaries using Bibby Stuart Scientific SMP3 apparatus (Stuart Scientific, UK) and are uncorrected. The IR spectra were recorded on FT-IR (PerkinElmer, USA) spectrophotometer using KBr disks. ^1^ H-NMR spectra were recorded using a Bruker 400 (Rheinstatten, Germany) spectrometer, and chemical shifts are expressed as *δ* (ppm) with tetramethylsilane as internal standard. The MS spectra were obtained with a Finnigan MAT TSQ-70 spectrometer (Finnigan MAT, Germany).

#### General procedure for the synthesis of 3-azolyl-4-chromanone derivatives 2a-c

Compounds **2a-c** was synthesized from 2-azolyl-2'-hydroxyacetophenones **1a-c** according to the literature methods [6-8].

#### General procedure for the synthesis of phenyhydrazone derivatives 3a-c

Phenylhydrazine hydrochloride was added to a solution of compound **2** (0.5 mmol) in methanol (4 mL) and the solution was stirred at room temperature. The completion of the reaction was checked by TLC. After consumption of compound **2** (3–7 days), water was added and the mixture was left in refrigerator overnight. The precipitated solid was separated by filtration and washed with water. The product was re-crystallized from methanol.

*2,3-Dihydro-3-(1H-1,2,4-triazol-1-yl)-4H-1-benzopyran-4-one phenylhydrazone* (**3a**)

Yield 55 %; IR (KBr, cm^-1^) 3418, 3238, 3128, 1703, 1603, 1561, 1497, 1478, 1459, 1278, 1261, 1216, 1138, 1055, 831, 763, 691, 678. ^1^ H NMR (400 MHz, DMSO-*d*_*6*_) δ 4.44 (m, 1 H), 4.82-4.87 (m, 1 H), 5.99 (dd, 1 H, *J* = 10.8 and 6.6 Hz), 6.79-6.90 (m, 2 H), 7.04 (t, 2 H, *J* = 7.2 Hz), 7.12-7.26 (m, 3 H), 7.66 (dt, 1 H, *J* = 7.4 and 2.0 Hz), 7.83 (dd, 1 H, *J* = 7.6 and 2.0 Hz), 8.43 (s, 1 H), 8.64 (s, 1 H), 10.05 (s, 1H). Anal. Calcd for C_17_H_15_N_5_O: C, 66.87; H, 4.95; N, 22.94. Found: C, 66.98; H, 5.14; N, 22.71.

*trans-2,3-Dihydro-3-(1H-imidazol-1-yl)-2-methyl-4H-1-benzopyran-4-one phenylhydrazone* (**3b**)

Yield 62%; m.p. 228–230°C; IR (KBr, cm^-1^) 3444, 3246, 1603, 1567, 1498, 1457, 1295, 1255, 1220, 1124, 834, 750, 696. ^1^ H NMR (400 MHz, DMSO-*d*_*6*_) δ 1.16 (d, 3 H, *J* = 6.4 Hz), 5.19 (dq, 1 H, *J* = 12.4 and 6.4 Hz), 5.88 (d, 1 H, *J* = 12.8 Hz), 6.75 (d, 2 H, *J* = 7.2 Hz), 6.83 (t, 1 H, *J* = 8.0 Hz), 6.92 (t, 2 H, *J* = 8.0 Hz), 7.01 (d, 1 H, *J* = 8.0 Hz), 7.11 (t, 1 H, *J* = 7.6 Hz), 7.14-7.36 (m, 3 H), 7.68 (t, 1 H, *J* = 8.4 Hz), 8.07 (d, 1 H, *J* = 7.6 Hz), 10.34 (s, 1 H). Anal. Calcd for C_19_H_18_N_4_O: C, 71.68; H, 5.70; N, 17.60. Found: C, 71.50; H, 5.79; N, 17.86.

*7-Chloro-2,3-dihydro-3-(1H-imidazol-1-yl)-4H-1-benzopyran-4-one phenylhydrazone* (**3c**)

Yield 58%; m.p. 192–194°C; IR (KBr, cm^-1^) 3444, 1602, 1560, 1497, 1418, 1252, 1163, 1082, 814, 754, 696. MS (*m/z*, %) 338 (M^+^, 25), 270 (100), 207 (10), 154 (15), 105 (28), 77 (78), 67 (37). Anal. Calcd for C_18_H_15_ClN_4_O: C, 63.81; H, 4.46; N, 16.54. Found: C, 63.66; H, 4.28; N, 16.57.

### Antifungal activity

The antifungal activities of title compounds **3a-c** and **2b** were tested against several representative pathogenic fungi including *Candida albicans* PTCC 5027 and *Saccharomyces cerevisiae* PTCC 5177 (as yeast), *Microsporum gypseum* PTCC 5070 (as a dermatophyte) and *Aspergillus niger* PTCC 5012 (as a mould) using agar dilution method [12]. Stock solutions of tested compounds were prepared in DMSO and serially diluted. Sabouraud Dextrose Agar was used for fungal growth. Inocula containing approximately 10^5^ CFUs/mL of fungi were prepared from broth cultures in log phase growth. Fungal plates were made in triplicate and incubated at 30°C about 24–48 h for yeast, about 72 h for moulds and about 168 h for dermatophytes. The MIC value was defined as lowest concentration of the antifungal agent at which the test strain does not demonstrate visible growth. The reported MICs are representative of at least two independent experiments.

### Docking study

Crystal structure of cytochrome P450 14α-sterol demethylase (Cyp51) from *Mycobacterium tuberculosis* in complex with fluconazole (ID 1EA1) was provided from the Protein Data Bank and prepared for docking calculations using AutoDock Tools 1.5.4 with default settings. Docking was performed using AutoDock vina,and standard parameters were used. Water molecules were not included. The binding site was defined to any residues with 10 Å distant from the co-crystallized fluconazole in the complex crystal structure of the enzyme [13].

### In silico drug-likeness and toxicity predictions

OSIRIS Property Explorer [14] was used to estimate the risks of side effects, such as mutagenic, tumorigenic, irritant and reproductive effects, as well as drug-relevant properties including cLog*P*, Log*S* (solubility), MW, drug-likeness and overall drug-score.

## Results and discussion

### Chemistry

**Scheme 1:**
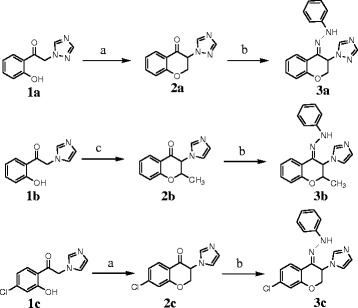
**Synthetic routes to phenylhydrazone derivatives 3a-c.** (**a**) paraformaldehyde, AcOH, reflux; (**b**) phenylhyrazine hydrochloride, MeOH, r.t.; (**c**) acetaldehyde, AcOH, 90°C.

The synthetic route to phenylhydrazone derivatives **3a-c** was illustrated in Scheme 1. The starting 2-azolyl-2'-hydroxyacetophenones **1a-c** was prepared from corresponding 2-bromo-2'-hydroxyacetophenones according to the literature method [15]. Cyclization of compounds **1a-c** with paraformaldehyde or acetaldehyde in refluxing acetic acid gave 3-azolyl-4-chromanones **2a-c**[6-8]. The synthesis of hydrazone derivative **3a** was first attempted by reaction of 3-triazolyl-4-chromanone **2a** with phenylhydrazine hydrochloride in refluxing methanol in the presence of Na_2_CO_3_ as a base, which resulted in poor yield with mixture of unknown degraded products. Since the condensation reactions of amines and hydrazines with ketones are commonly catalyzed by acids or bases, thus the alternative condition was using hydrochloride salt of phenylhydrazine in the absence of a base. Accordingly, the reaction of compound **2a-c** with phenylhydrazine hydrochloride in methanol at room temperature afforded final compounds **3a-c** in a mild condition. It seems that the acid released by hydrochloride salt of phenylhydrazine is adequate for catalyzing the condensation reaction.

**Scheme 2:**
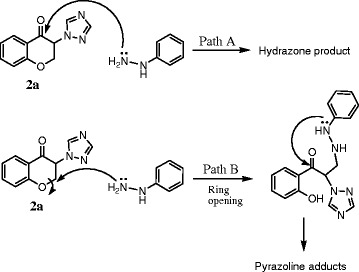
Possible reactivity of 3-triazolylchroman-4-one derivative 2a toward phenylhydrazine.

Theoretically, the reactivity of chromanone derivatives toward N-nucleophiles like substituted hydrazine related to two sites; carbonyl group and C-2 position (Scheme 2). The attack of hydrazine to the C-2 position of chroman ring could result in ring opening and subsequent pyrazoline formation.

Previously, the reaction of chromanone and flavanone derivatives with substituted hydrazines has been investigated in different conditions [16,17]. Kállay et al. have been reported that the flavanone hydrazones are predominantly obtained under acidic conditions while alkaline conditions give the pyrazolines and the 2-hydroxychalcone derivatives due to the ring cleavage of the hetero ring of chromanone [16]. Similarly, in our experiment with 3-azolyl-4-chromanones, the hydrazone derivatives were predominantly obtained under mild acidic conditions, but in the presence of weak base Na_2_CO_3_, possibly produced a mixture of corresponding hydrazone, acyclic *N*-substituted hydrazine and pyrazoline derivatives which was difficult to separate and characterize (Scheme 2).

The structures of compounds **3a-c** were properly characterized by IR, ^1^ H NMR or Mass spectroscopy as well as elemental analyses. For example, elemental analysis proved the molecular formula of compound **3b** as C_19_H_18_N_4_O. The IR spectra of compound **3b** did not show any absorption due to the presence of C = O group and a broad band was found in 3444 cm^-1^ due to the presence of a N − H bond. The ^1^ H NMR spectrum of **3b** showed a singlet at 10.34 for NH, a double at 8.07 (*J* = 7.6 Hz) and a triplet at 7.68 (*J* = 8.4 Hz) for H-5 and H-7 of chroman ring, respectively. The resonance for imidazole protons was observed as a multiplet at the range of 7.14-7.39 ppm. The H-6 and H-8 of chroman ring resonated as a triplet at 7.11 and a doublet at 7.01 ppm. Five protons related to the phenyl group appeared at 6.92, 6.83 and 6.75 ppm. The aliphatic protons of chroman (H-3 and H-2) resonated as a doublet at 5.88 and a doublet of quartet at 5.19 ppm. A doublet appeared at 1.16 ppm, assigned to the methyl protons.

### Antifungal activity

The antifungal activities of title compounds **3a-c** were tested against several representative pathogenic fungi. The resulting MICs (minimum inhibitory concentrations) of hydrazone compounds were compared with those of fluconazole as a reference antifungal agent and intermediate compound **2b** (Table 1). The results revealed that 2-methyl-3-imidazolylchroman-4-one (**2b**) showed no activity against *Saccharomyces cerevisiae*, *Aspergillus niger* and *Microsporum gypseum* (MIC >64 μg/mL). Its activity against *Candida albicans* was weak. Compounds **3a,b** exhibited good growth inhibitory activity against different fungal strains with MIC values of 8–16 μg/mL. The 7-chloro-3-imidazolyl derivative **3c** also showed good antifungal activity against *Candida albicans*, *Saccharomyces cerevisiae* and *Microsporum gypseum* (MIC = 16 μg/mL), but its activity against *A. niger* was moderate. The MIC values of fluconazole against test strains were 8–32 μg/mL. Thus, the antifungal activity of title compounds was comparable to that of standard drug fluconazole. By comparing the MIC values of hydrazone derivative **3b** and its ketone counterpart **2b**, it is revealed that the introduction of phenylhydrazone on chroman ring improves the antifungal activity against all strains.

**Table 1:** ***In vitro *antifungal activity of compounds 3a-c in comparison with 2b and fluconazole**

***Compound***	**Tested fungi (MICs in μg /mL)**			
	***C. albicans***	***S. cerevisiae***	***A. niger***	***M. gypseum***
**2b**	64	>64	>64	>64
**3a**	16	16	16	8
**3b**	8	16	16	16
**3c**	16	16	64	16
Fluconazole	8	32	32	32

Previously, we have described azolylchromanone oxime ethers as conformationally restricted analogs of oxiconazole (Figure 1). Many of these derivatives exhibit high activity against different fungal strains [6-8]. In this work, we replaced *O*-benzyloxime ether fragment in azolylchromanone derivatives by phenylhydrazone moiety. The comparison of MIC values of triazole derivative **3a** with its *O*-benzyloxime ether counterpart demonstrates that replacement of *O*-benzyloximino moiety with hyrazone can increase antifungal potency. However, in the cases of **3b** and **3c**, their *O*-benzyloximino analogs have a better profile of antifungal activity.

### Docking study

Azole antifungals such as fluconazole inhibit fungal cytochrome P-450 dependent 14α-demethylase [4]. With the aim of rationalizing the antifungal activity data obtained, docking study was performed for the selected derivatives **2b** and **3a** in order to investigate the possible interactions with cytochrome P450 14α-sterol demethylase from *Mycobacterium tuberculosis* (Mycobacterium P450 DM). Because no experimental data are available for the target enzyme Candida P450 DM in the Protein Data Bank, the crystallographic structure of the complex between Cytochrome P450 14α-sterol demethylase from *Mycobacterium tuberculosis* (Mycobacterium P450 DM) and fluconazole (ID 1EA1) was used for the docking study [18].

**Figure 3:**
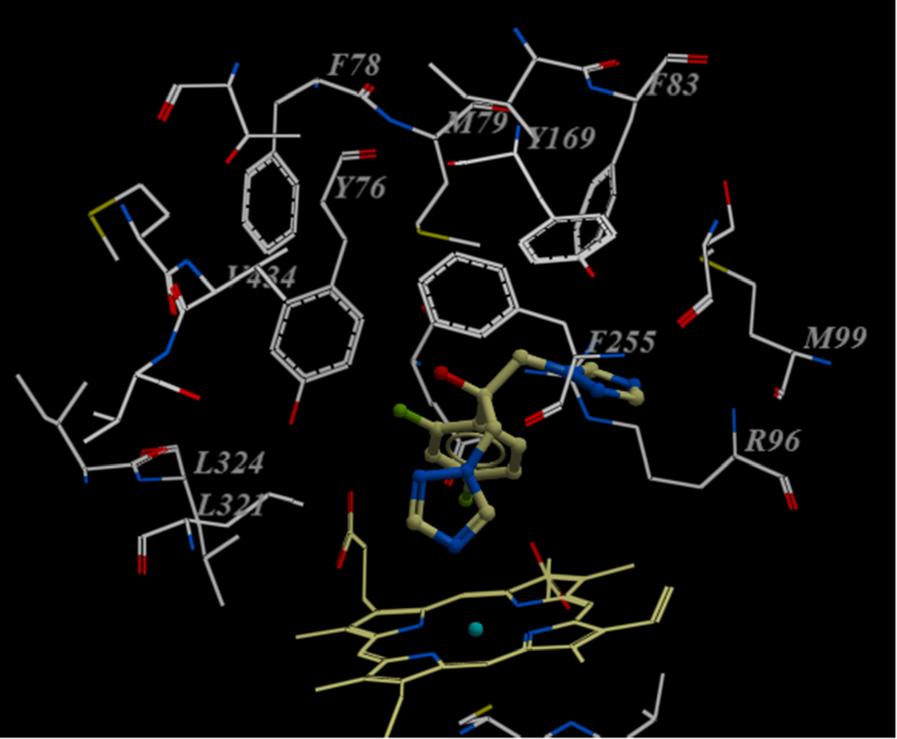
Computed binding geometry of the reference compound fluconazole (ball and stick) in the active site of Mycobacterium P450DM.

**Figure 4:**
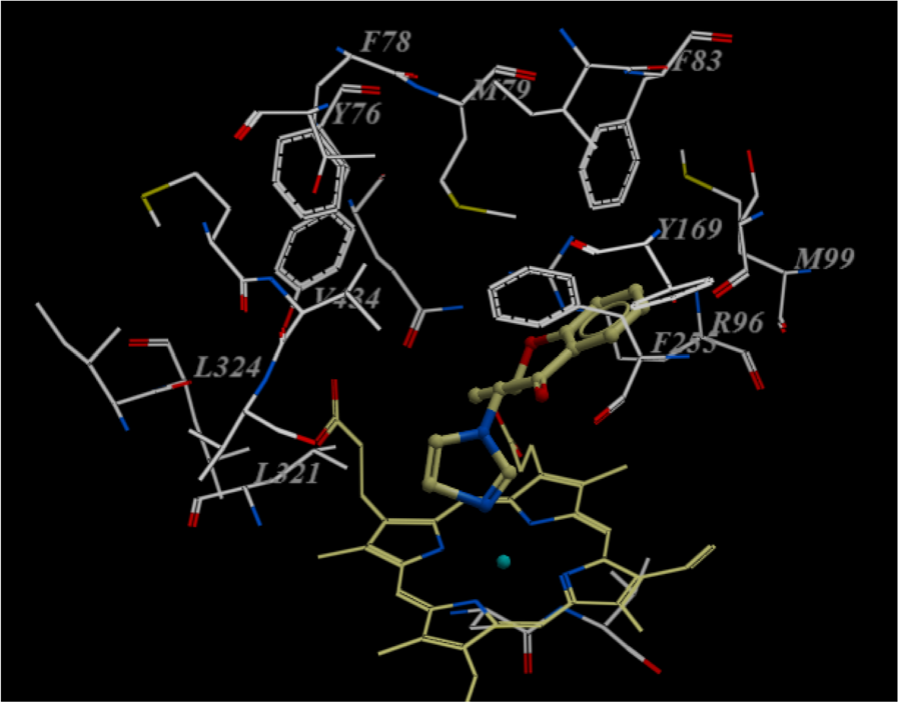
**Compound 2b (ball and stick) in the active site of Mycobacterium P450 DM. **For clarity, only the amino acids with 10 Ǻ distant from the compound **2b** are shown.

**Figure 5:**
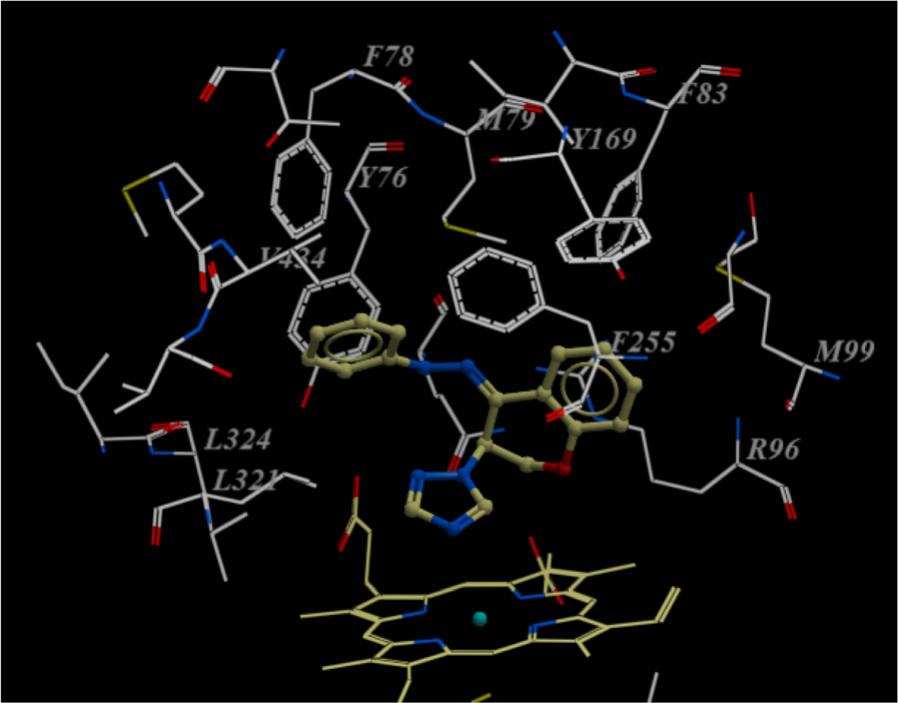
**Compound 3a (ball and stick) in the active site of Mycobacterium P450 DM. **For clarity, only the amino acids with 10 Ǻ distant from the compound **3a** are shown.

Figure 3 shows the docked reference compound fluconazole in the active site of the same enzyme for validation of our docking protocol and confirmation of the biological data. Figures 4 and 5 show complex between the enzyme's active site and compounds **2b** and **3a**, respectively. The aromatic part of chroman ring of the both compounds is situated in a lipophilic region and interacts with Phe255, Met99, Phe83 and Met79. Oxygen atom (sp^3^ hybridized) of the chroman ring is oriented towards the polar region of the enzyme constituted by Arg96 and Arg95. The phenyl attached to the hydrazone moiety is inserted into a hydrophobic pocket above the heme ring formed by ceiling residues Met79, Phe78, Phe255, Val434, Leu321 and Tyr76. The azole ring of both compounds **2b** and **3a** are positioned above the porphyrine plan, with a ring nitrogen atom coordinated to the heme iron. The distance between nitrogen atom of compounds **2b** and **3a** and the heme iron is 2.6 Å and 2.8 Å, respectively.

It has been previously reported that hydrophobic interactions with Val434 is responsible for the potent inhibitory activity of antifungals against *Aspergillus niger* whereas hydrophobic interactions with Leu321, Ile323, Leu324 may be responsible for an enhanced inhibitory activity against *Candida albicans* and other Candida spp. [18]. As shown in Figures 4 and 5, these interactions only can be seen with compound **3a** prototype bearing lipophilic phenylhydrazone side chain and not with compound **2b**; explaining the major potency differences observed in obtained results between **2b** and compounds **3ac**.

### In silico drug-likeness and toxicity predictions

Currently, several approaches have been developed to assess drug-likeness of bioactive compounds based on topological descriptors, fingerprints of molecular structure or other properties such as molecular weight, water solubility and cLog*P*[19]. In this work, open-source program OSIRIS Property Explorer [14] was used to predict the fragment-based drug-likeness of title compounds and comparing them with fluconazole. OSIRIS program involves the database of traded drugs and commercially available compounds (Fluka) assumable as non drug-like dataset to assess the occurrence frequency of each fragment in the individual structure. The program estimated the risks of side effects, such as mutagenic, tumorigenic, irritant and reproductive effects, as well as drug-relevant properties including cLog*P*, Log*S* (solubility), MW and drug-likeness. Moreover, the overall drug-score was estimated by combining outcome of cLog*P*, Log*S*, MW, toxicity risks and drug-likeness. Drug-score is a measure of compound's potential to meet the criteria of a possible drug candidate [14].

The *in silico* drug-relevant properties obtained by OSIRIS Property Explorer are given in Table 2. Interestingly, the potential drug-likeness values of compounds **3a-c** were significantly higher than that of fluconazole, which showed a negative value of −1.13. As shown in Table 2, compounds **3a-c** had high *in silico* tumorigenic and medium mutagenic toxicity risks due to the phenylhydrazone moiety. However, the *in silico* prediction by OSIRIS Property Explorer showed that the introduction of chlorine on phenylhydrazone can reduce the risk of tumorigenic and mutagenic toxicities. Generally, the drug-score values of compounds **3a-c** (0.2-0.34) were less than that of fluconazole (0.46). Among the final compounds, 2-methyl-3-imidazolyl derivative **3b** showed the highest values of drug-likeness and drug-score.

**Table 2:** Toxicity risks and physicochemical properties of compounds 3a-c in comparison with fluconazole, predicted by OSIRIS Property Explorer

**Compound**	**Toxicity risks**				**Physicochemical properties**				
	**Mutagenic**	**Tumorigenic**	**Irritant**	**Reproductive effect**	**CLogP**	**Slobility**	**MW**	**Drug likeness**	**Drug-score**
**3a**	(±)	(+)	(−)	(±)	4.18	−3.07	305	1.33	0.26
**3b**	(±)	(+)	(−)	(−)	4.46	−2.78	318	2.81	0.34
**3c**	(±)	(+)	(−)	(±)	4.79	−3.14	338	0.17	0.2
**Flu**	(−)	(−)	(−)	(±)	−0.21	−2.17	306	−1.13	0.46

## Conclusion

In conclusion, a series of 3-azolyl-4-chromanone phenylhydrazones were synthesized via ring closure of 2-azolyl-2'-hydroxyacetophenones and subsequent reaction with phenylhydrazine. These compounds considered as analogs of 3-azolyl-4-chromanone oxime ethers basically designed as antifungal agents. The *in vitro* antifungal activity of compounds based on MIC values revealed that all compounds showed good antifungal activity against *Candida albicans*, *Saccharomyces cerevisiae* and *Microsporum gypseum* at concentrations less than 16 μg/mL. The antifungal activity of title compounds was comparable to that of standard drug fluconazole. The biological data and docking study demonstrated that the introduction of phenylhydrazone moiety on chroman ring improves the antifungal activity. The 2-methyl-3-imidazolyl derivative **3b** which showed the highest drug-likeness and drug-score values serves as a lead for future development of antifungal agents.

## Competing interests

The authors declare that they have no competing interests.

## Authors’ contribution

AA participated in the synthesis of compounds. MF performed antifungal activity evaluation. HI participated in the molecular modeling study and performed the docking analysis. SE participated in the design of compounds, supervision of synthetic and computational parts, identifying of the structures of target compounds, accomplishing drug-likeness predictions and manuscript preparation. All authors read and approved the final manuscript.
